# Extremely high doses of radioiodine required for treatment of Graves’ hyperthyroidism: a case report

**DOI:** 10.4076/1757-1626-2-8479

**Published:** 2009-08-25

**Authors:** Arnaldo Moura Neto, Marcos Antonio Tambascia, Sergio Brunetto, Celso Dario Ramos, Denise Engelbrecht Zantut-Wittmann

**Affiliations:** 1 Endocrinology Division, Internal Medicine Department, Medical Science School, University of CampinasCampinas, S.P.Brazil; 2 Nuclear Medicine Division, Radiology Department, Medical Science School, University of CampinasCampinas, S.P.Brazil

## Abstract

**Introduction:**

Radioactive iodine (^131^I) is widely prescribed for treatment of Graves’ disease. A dose of 370 to 555 MBq (10 to 15 mCi) is usually enough, but reports of improved remission rates with single doses up to 20-30 mCi, and 38.5 mCi at most, exist.

**Case presentation:**

A 53-year-old male patient was evaluated in September 2005, with symptoms of thyrotoxicosis for 2 years. He presented with tachycardia (130 bpm) and a large goiter. Thyrotropin was <0.01 uIU/ml (0,41-4,5), free thyroxin >7.77 ng/dl (0.9-1.8), anti-thyreoperoxidase antibody: 374 IU/ml (<35) and anti-thyroglobulin antibody: 749 IU/ml (<115). Ultrasound: diffuse goiter, no nodules; right lobe: 7.9 × 3.8 × 3.8 cm; left: 7.7 × 3.5 × 3.8 cm; isthmus: 1.6 cm. Propylthiouracil 300 mg t.i.d. and propranolol were prescribed. Thyroid ^99m^Tc-pertechnetate uptake: 52% (0.35-1.7%) and estimated thyroid volume: 149 mL. After 30 days, he received 555 MBq (15 mCi) of ^131^I-iodide. Six months after radioiodine therapy, under methimazole 40 mg, thyroid stimulating hormone was 1.5 uIU/ml; free thyroxine 0.54 ng/dl. Methimazole was suspended. In 21 days, thyroid stimulating hormone was 0.03 uIU/ml; free thyroxine 0.96 ng/dl. Methimazole was reintroduced. One year later, thyroid stimulating hormone was <0.01 uIU/ml and free thyroxine >7.77 ng/dl. Thyroid ^99m^Tc-pertechnetate uptake was 45% and estimated thyroid volume 144 mL. A 1110 MBq (30 mCi) radioiodine therpy was administered. He used Methimazole for 8 months, when overt hypothyroidism appeared (TSH: 25.30 uIU/ml; free thyroxine: 0.64 ng/dl). Methimazole was interrupted. Hyperthyroidism returned 6 weeks later (thyroid stimulating hormone <0.01 uIU/ml; free thyroxine >7.77 ng/dl). Thyroid ^99m^Tc-pertechnetate uptake was 25% and estimated thyroid volume 111 mL. Methimazole was prescribed again. In March 2008 he received a 2590 MBq (70 mCi) radioiodine therapy. By may/2008, under methimazole 20 mg, his TSH was 0.07 uIU/ml; free thyroxine 1.31 ng/dl. In October 2008 he presented overt hypothyroidism (TSH 91.6 uIU/ml; free thyroxine 0.34) and was given levothyroxine 75 mcg/day. He remains euthyroid under hormone replacement.

**Conclusion:**

Our presented case of Graves’ disease received a cumulative dose of 4255 MBq (115 mCi). The high uptake could indicate accelerated iodine turnover with ^131^I short time of action. Impaired hormone synthesis could also be present. We believe the extremely high dose required was due to the initial very high iodine uptake and large thyroid volume.

## Introduction

Graves’ disease is the most common cause of hyperthyroidism worldwide. The administration of radioactive iodine (^131^I) has become widely prescribed for treatment of this condition, because of both its safety and easiness of use [1]. Much controversy has aroused as regard to the best fixed dose sufficient to relieve the patient from hyperthyroidism. Some authors advocate the use of higher doses, intended to deliberately cause hypothyroidism soon after treatment, and in so doing reduce the incidence of failure and relapse. Usually, a dose of 370 to 555 MBq (10 to 15 mCi) is considered enough for this purpose [2], but there are some reports of improved remission rates with up to 740-1110 MBq (20-30 mCi) [3]. The majority of patients require only a single dose and euthyroidism is achieved in 6-8 weeks in 40 to 60% of them [4].Several factors have been studied as outcome predictors in the use of radioiodine therapy (RIT) for Graves’ disease, such as sex, age, thyrotropin (TSH) and auto-antibodies levels, thyroid volume and presence of ophthalmopathy [5]. Failure of RIT is mostly associated with larger goiter volume or size, and some studies recommend that a higher dose should be prescribed for these patients [6].

Generally, fewer than 10% of cases will require more than one dose [7]. Similarly, recurrences are rare after a hypothyroid state is achieved [8]. Reviewing the literature, we found one report of a patient presenting relapse of Graves’ hyperthyroidism 10 years after receiving a dose of 1424.5 MBq (38.5 mCi) [9], and none requiring more for management of Graves’ disease.

## Case presentation

We present the case of a, 53 years old male, Caucasoid patient, attended at our Endocrinology service in September 2005, proceeding from an iodine sufficient area. His main complaint was cervical enlargement for 2 years, associated with weight loss (9 kg), tremors, palpitations and fatigue. He denied any previous health conditions such as hypertension or Diabetes Mellitus and was a non-smoker. On physical examination he presented tachycardia (130 bpm), an arrhythmic pulse and hypertension (160 × 100 mmHg). Thyroid palpation revealed a large goiter with firm consistency, smooth surface, preserved mobility and no murmur on auscultation. There was no apparent ophthalmopathy.

Laboratory tests showed: TSH: <0.01 uIU/ml (0,41-4.5); FT4: >7.77 ng/dl (0.9-1.8) and T3: >651 ng/dl (80-200). Anti-thyreoperoxidase antibody: 374 IU/ml (<35); Anti-thyreoglobulin antibody: 749 IU/ml (<115); Complete Blood Count: White Blood Cells: 4030/mm3; Hemoglobin: 13.2 g/dl; Hematocrit: 38,1% and 219,000 platelets/mm^3^. EKG demonstrated atrial fibrillation with a ventricular response of approximately 140 bpm. Thyroid ultrasound showed diffuse goiter with hypoechogenic parenchyma and no nodules. Right lobe: 7.9 × 3.8 × 3.8 cm; left lobe 7.7 × 3.5 × 3.8 cm; isthmus 1.6 cm. Transthoracic echocardiogram revealed an ejection fraction (EF) of 61% with mild enlargement of the left atrium and also mild incompetence of all four cardiac valves. Estimated peak pulmonary systolic pressure was 51 mmHg, compatible with mild pulmonary hypertension. Therefore, the preliminary diagnosis was that of Grave’s disease with atrial fibrillation.

**Figure 1.**
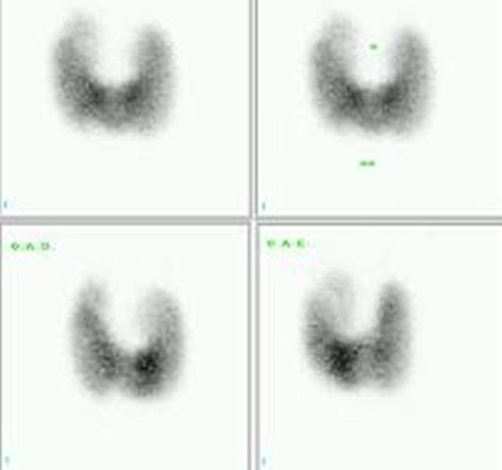
Patient’s first thyroid scan showing a 52% ^99m^Tc-pertechnetate uptake and estimated volume of 149 mL.

It was decided for admission in order to attain better clinical management and close monitoring. Propylthiouracil (PTU) 300 mg t.i.d., along with propranolol and warfarin were prescribed. During the patient’s stay there was spontaneous reversion to sinus rhythm. He was discharged 11 days later and in one month, October/2005, received a 555 Mbq (15 mCi) oral dose of ^131^I. Thyroid scan previous to radioiodine administration showed diffuse goiter and a ^99m^Tc-pertechnetate uptake of 52% (0.35-1,7%). Using planar ^99m^Tc-pertechnetate images, thyroid volume estimated from this scan was 149 mL (Figure 1). PTU was interrupted after the radioiodine treatment (RIT) even though the patient was still complaining of palpitations. Ten days later, laboratory workup was: TSH: 0.01 uIU/ml; FT4: >7.77 ng/dl. He was kept in use of propranolol 80 mg tid and methimazole (MMI) 40 mg q.d. was initiated.

Six months after RIT, the patient’s TSH level was 1.5 uIU/ml; FT4 0.54 ng/dl and FT3 4.32 pg/ml (2.57-4.43). MMI was suspended at this point, as the patient was asymptomatic. Twenty days after discontinuation of MMI, however, TSH was 0.03 uIU/ml, FT4 0.96 ng/dl and FT3 0.51 ng/dl (0.257-0.443) and MMI was reintroduced.

One year after RIT, the patient relapsed into persistent hyperthyroidism and second RIT was scheduled. TSH was <0.01 uIU/ml, FT4 > 7.77 ng/dl and FT3 2.07 ng/dl. A new thyroid scan demonstrated diffuse goiter and 45% ^99m^Tc-pertechnetate uptake (estimated volume of 144 mL). Echocardiogram showed EF of 70% and mild mitral and tricuspid regurgitation. A RIT of 1110 MBq (30 mCi) was administered in February 2007. The patient was kept in use of MMI until 8 months after the second RIT, when he developed overt hypothyroidism (TSH: 25.30 uIU/ml; FT4: 0.64 ng/dl). However, relapse of hyperthyroidism occurred only 45 days after MMI withdrawal (TSH < 0.01 uIU/ml; FT4 > 7.77 ng/dl). A third scan was performed, revealing 25% ^99m^Tc-pertechnetate uptake (estimated volume of 111 mL). Consequently, MMI was again prescribed, and a third RIT planned.

In March 2008 he was admitted for 36 hours in order to receive a dose of 2590 MBq (70 mCi) of ^131^I. Three months later, the use of MMI 20 mg q.d. was still required and his TSH level was 0.07 uIU/ml, FT4: 1.31 ng/dl and FT3: 0.30 ng/dl.

In October 2008, his laboratory workup showed overt hypothyroidism (TSH 91.6 uIU/ml and FT4 0.34). MMI was withdrawal but hypothyroidism persisted and he was therefore put on levothyroxine 75 mcg q.d. Six weeks later, euthyroidism was restored (TSH: 0.51 uIU/ml and FT4: 1.78 ng/dl). He remains euthyroid with levothyroxine replacement until the present date and no dose change was required so far.

## Discussion

Our presented case of Graves’ hyperthyroidism received a cumulative ^131^I treatment of 4255 MBq (115 mCi). After the first two RIT, the patient presented transient overt hypothyroidism and rapidly relapsed into hyperthyroidism after discontinuation of anti-thyroid therapy. Even three months after the third higher dose, and in use of 20 mg of MMI, he remained with suppressed TSH levels. Only five months after the last RIT would lasting hypothyroid state manifest, requiring levothyroxine treatment. We found no case reports in the literature of such a high ^131^I requirement to control Graves’ disease.

Transient or definitive hypothyroidism after a radioiodine dose is common, due to the acute injury to thyroid cells and incapacitation to maintain the high hormone synthesis [10]. In addition, less than 15% of cases will relapse into hyperthyroidism and require a second dose. The need for more than two doses is even rarer [9].

**Figure 2.**
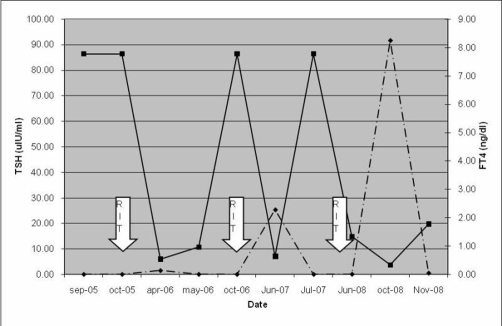
Thyroid function Patient’s TSH (dotted line) and FT4 (solid line) from Sep-05 to Nov-08. ^131^I (RIT) was administered in Oct-05, Oct-06 and Mar-08.

The reason for the atypical behavior of this case can be at least partially explained by the initial high ^99m^Tc-pertechnetate uptake found in the first scan. The radiopharmaceutical ^99m^Tc-pertechnetate uptake reliably reflects thyroid avidity for iodide. In Graves’ disease there is an elevated clearance of plasma iodide and increased thyroid peroxidase activity, leading to a higher hormone synthesis. Therefore iodine turnover is faster and consequently the residence time of radioiodine within the gland is shorter. This could culminate in a reduced efficacy of RIT (Figure 2) [11].

Supporting this hypothesis is also the very short period of time during which the patient remained in hypothyroidism following the first two RIT, possibly indicating a quick, not sustained action of ^131^I on thyroid cells because of its rapid turnover. Although not curative, they reduced ^99m^Tc-pertechnetate uptake from 52% to 45%, and then to 25%, making it more likely that another RIT would be definitive and also demonstrating the partial effect of the ^131^I administered in these first two treatments. A very high, 2590 MBq (70 mCi) third RIT was then administered hoping that a higher ^131^I dose would compensate for its shorter time of action.

An impaired hormone synthesis could also be considered to explain this very high uptake, but is much less likely because of the high thyroid hormone levels at the time of diagnosis and also throughout follow up.

Additionally, large goiters are associated with higher incidence of failure and relapse [6] and maybe this is the key factor in this case. When a fixed dose approach is used, small and medium-sized glands have high and similar cumulative incidence of hypothyroidism or euthyroidism following radioiodine treatment. Larger glands receiving the same dose, however, tend to have a higher rate of persistent hyperthyroidism after 1 year. The difference in success rates is even more apparent in the first 6 months [6]. Therefore for patients with larger goiter and higher ^99m^Tc-pertechnetate uptakes, a calculated dose based on size and uptake is perhaps a better decision [12]. Surgical treatment (partial thyroidectomy) is also a reasonable option in such cases, as it propitiates prompt relief of symptoms related to compression of the esophagus and/or trachea [13].

In the presented case, the first RIT with 555 MBq (15 mCi) may not have been sufficient to cause complete ablation of thyroid cells, because of the patient’s increased gland volume. Residual cells viable and under continuous stimulation of thyroid-stimulation immunoglobulin could have caused recurrent hyperthyroidism. A similar fact must have occurred after administration of the 1110 MBq (30 mCi) second RIT.

## Conclusion

In conclusion, although the use of a fixed dose method simplifies the approach to treatment with potential cost savings, we believe that a higher dose of RIT should be considered for patients with Graves’ disease presenting an initial very high thyroid ^99m^Tc-pertechnetate uptake. This is especially true when high uptake is associated with increased goiter size, as failure and relapse in such cases are more likely to occur, leading to increased morbidity and the need for further radiation exposure.

## Abbreviations

EF: ejection fraction
FT3: free triiodotironine
FT4: free thyroxine
MMI: methimazole
PTU: propylthiouracil
RIT: radioiodine therapy
T3: total triiodotironine
TSH: thyroid stimulating hormone
